# Longitudinal Outcomes in Octogenarian Critically Ill Patients with a Focus on Frailty and Cardiac Surgery

**DOI:** 10.3390/jcm10010012

**Published:** 2020-12-23

**Authors:** Aileen Hill, Daren K. Heyland, Rolf Rossaint, Rakesh C. Arora, Daniel T. Engelman, Andrew G. Day, Christian Stoppe

**Affiliations:** 1 Department of Intensive Care Medicine, University Hospital RWTH, D-52074 Aachen, Germany; 2 3CARE—Cardiovascular Critical Care & Anesthesia Evaluation and Research, D-52074 Aachen, Germany; 3 Clinical Evaluation Research Unit, Department of Critical Care Medicine, Queen’s University, Kingston General Hospital, Kingston, ON K7L 2V7, Canada; dkh2@queensu.ca; 4 Department of Anesthesiology, University Hospital RWTH, D-52074 Aachen, Germany; rrossaint@ukaachen.de; 5 Section of Cardiac Surgery, Department of Surgery, Max Rady College of Medicine, Winnipeg, MB R2H 2A6, Canada; rarora@sbgh.mb.ca; 6 Heart and Vascular Program, Baystate Health, Medical School-Baystate, University of Massachusetts, Springfield, MA 01199, USA; Daniel.engelman@baystatehealth.org; 7 KGH Research Institute, Kingston Health Sciences Centre, Kingston, ON K7L 2V7, Canada; Andrew.Day@Kingstonhsc.ca; 8 Department of Anesthesiology, Intensive Care Medicine and Pain Therapy, University Hospital Würzburg, 97080 Würzburg, Germany

**Keywords:** population characteristics, demography, aged 80 and over, critical illness, cardiac surgery, critical care, frailty, prospective studies, nutrition therapy

## Abstract

Cardiac surgery (CSX) can be lifesaving in elderly patients (age ≥ 80 years) but may still be associated with complications and functional decline. Frailty represents a determinant to outcomes in critically ill patients, but little is known about its influence on elderly CSX-patients. This is a secondary exploratory analysis of a multi-center, prospective observational cohort study of 610 elderly patients admitted to the ICU and followed for one year to document long-term outcomes. CSX-ICU-patients (*n* = 49) were compared to surgical ICU patients (*n* = 184) with regard to demographics, frailty, and outcomes. Of all surgical patients, 102 (43%) were considered vulnerable or frail. The subdistribution hazard ratio (SHR) of time to discharge home (TTDH) for vulnerable/frail vs. fit/well patients was 0.54 (95% confidence interval (CI), 0.34, 0.86, *p* = 0.007). The *p*-value for effect modification between surgery group (CSX vs. surgical ICU patients) and Clinical Frailty Scale (CFS) group was not significant (*p* = 0.37) suggesting that the observed difference in the CFS effect between the CSX and surgical ICU patients is consistent with random error. A further subgroup analysis shows that among surgical ICU patients, the SHR of time to discharge home (TTDH) for vulnerable/frail vs. fit/well patients was 0.49 (95% CI, 0.29, 0.83) while the corresponding SHR for CSX patients was 0.77 (0.32–1.88). In conclusion, preoperative frailty reduced the rate of discharge to home in both surgical and CSX patients, but a larger sample of CSX patients is needed to adequately address this question in this patient group.

## 1. Introduction

An increasing number of elderly patients (age ≥ 80 years) undergo complex and invasive surgeries [1]. Among these major surgeries, cardiac surgery rates in this group will further increase in the future as it is increasingly performed on an older and comorbid patient population with complex coronary lesions [2]. These procedures can be mandatory to save the patient’s life and to improve their quality and quantity of life [3]. However, despite substantial advances in the medical treatment and the option to perform minimally invasive interventions in some patients, open heart surgery may result in an acute and prolonged decline of physical function, especially in vulnerable patients [4,5]. Additionally, the frequent occurrence of postoperative organ dysfunction, perioperative complications and the resulting decrease in patients’ functional status may lead to prolonged intensive care unit (ICU)- and hospital-length-of stays (LOS), delayed rehabilitation, increased morbidity and mortality, and reduced health-related quality of life (HRQOL) [6,7] which may sometimes outweigh the overall benefit of the surgery itself. An improvement of the quality of life and the ability to live independently are often of greater importance to the elderly patient and their families, than the sole prolongation of life [8,9]. The overall high survival rates of about 92% after one year [10] and 70% after five-years [11], demonstrate the need to further explore potential risk factors influencing post-ICU quality of life, such as physical function and the possibility to return to home [12]. This becomes even more important after noting that only 25% of elderly patients admitted to an ICU return to their baseline physical function after one year [13].

Elderly patients differ from the general ICU or surgical population, because of higher incidences in comorbidities and frailty associated with ageing. Frailty is a multidimensional syndrome, where a loss of resources leads to increased vulnerability in patients with the inability to cope with stressor events, such as surgery [14,15]. Frailty has been shown to be a significant predictor of short and long-term clinical outcomes in the general ICU patient population [13,16]. These patients could represent targets for specific prehabilitation strategies, such as preoperative nutrition and physical therapy [17,18]. In addition, if the impact of frailty in elderly patients undergoing cardiac surgery were well-researched, frailty might be a criterion to recommend patients for either open heart surgery or less invasive percutaneous interventions.

Until now, the prevalence and impact of frailty on patients undergoing cardiac surgery has been described mainly in single center studies, limited patient selection to elective procedures, such as isolated coronary artery bypass graft or simple valve surgery and have not commonly focused on patients ≥ 80 years of age [5,19,20,21,22,23]. Our work therefore aims to describe the impact of frailty on elderly cardiac surgery patients in the ICU in comparison to elderly surgical ICU patients who did not undergo cardiac surgery. Surgical ICU patients of the same age provide a relevant comparator group that aids in the interpretation of our findings.

## 2. Methods

This multicenter, prospective observational cohort study was conducted from September 2009 until February 2013 in 24 Canadian ICUs (clinicaltrials.gov: NCT01293708). Institutional research ethics board approval was obtained from each center. Prior publications of this cohort study have focused on life-saving interventions provided [24], the clinical recovery of octogenarians [13], family member’s perspectives on clinical decision making [9], and cost analysis [25]. This is a secondary analysis comparing elderly patients undergoing cardiac surgery (CSX-group) with surgical ICU-patients. The Canadian Institutes of Health Research (CIHR) had no influence on the design of the study or the interpretation of the data.

### 2.1. Eligibility Criteria

All patients aged 80 years and older were eligible to be recruited for this trial if they remained in the ICU for at least 24 h, regardless of the primary diagnosis or if the ICU-admission was due to an emergency or after scheduled surgery. Patients included in our longitudinal analysis were followed up for 12 months, using interviews of patients or their caregivers.

Before enrollment, informed consent was obtained from the patient’s legal representative or participating family member, and subsequently from competent survivors. For this longitudinal cohort, exclusion criteria were (a) patient non-resident of Canada and (b) no family member available to complete data collection. Family members participating in this study were required to (a) visit the patient at least once during the first 96 h after ICU admission, (b) speak English or French, (c) give care without being paid to do so, and (d) be at least 18 years old. Patients were also excluded, if consent could not be obtained, or the patient was dying acutely.

In recognition of selection bias if only these patients were chosen, an additional hospital cohort was planned a priori, where ICU and hospital data were collected consecutively from all patients admitted to the participating ICUs who were not included in the longitudinal cohort. In the hospital cohort, data was obtained from chart review without requiring consent and will be presented as comparator group in Appendix A.

### 2.2. Data Collection

All information was collected by trained research personnel from key informants. Key informants were approached by the research staff to assess living location and frailty before hospital admission, and to ask for the patient’s documented will as well as the family’s preference for care. At baseline, the Sequential Organ Failure Assessment (SOFA) score and the Acute Physiology and Chronic Health Evaluation II (APACHE II) Score, the Charlson Comorbidity Index (CCI) [26] and the Clinical Frailty Scale (CFS) [14] were obtained. The CFS provides understanding of the patient’s level of fitness or frailty prior to hospital or ICU admission and ranges from 1 (very fit) to 7 (severely frail). The CFS questionnaire was initially completed with the closest family member or, if possible, by collecting the data directly from the patient later after they recovered. For ease of interpretation, we dichotomized the CSF as fit/well (scores 1–3) or vulnerable/frail (scores 4–7).

The primary outcomes of the parent study were the HRQOL (as measured by the physical domain and the physical component summary scale of the Short Form-36), functional status (as measured by the Palliative Performance Status Score), and survival and have been published elsewhere [13].

### 2.3. Statistical Analysis

This analysis aimed to compare critically ill elderly CSX-patients with elderly surgical ICU patients with regard to baseline demographics, processes of care and clinical outcomes. Continuous variables were described by means, standard deviations and ranges, except for the skewed length-of stay variables, CFS and CCI which were described by medians and quartiles. Categorical variables were described by counts and percentages. Group differences between elderly CSX and elderly surgical ICU patients or between fit/well and vulnerable/frail were tested by the Mann–Whitney U test for continuous variables and the chi-square test for the categorical variables.

Time to discharge to home (TTDH) was defined as the time from hospital admission to discharge home. Death or discharge to palliative care or a long-term care facility were considered competing risks precluding eventual discharge home, while discharge to another hospital, ICU, rehab or “other” were treated as censoring events. TTDH was depicted using cumulative incidence function curves showing the proportion of patients who returned home over time. The difference between groups in the cumulative incidence function curves was tested by Gray’s method and summarized by the sub-distribution hazards ratio (SHR) [27,28,29]. All tests are two-sided without correction for multiplicity. The analysis was performed using SAS version 9.4 (SAS Institute Inc., Cary, NC, USA).

## 3. Results

### 3.1. Patients Evaluated

**Figure 1:**
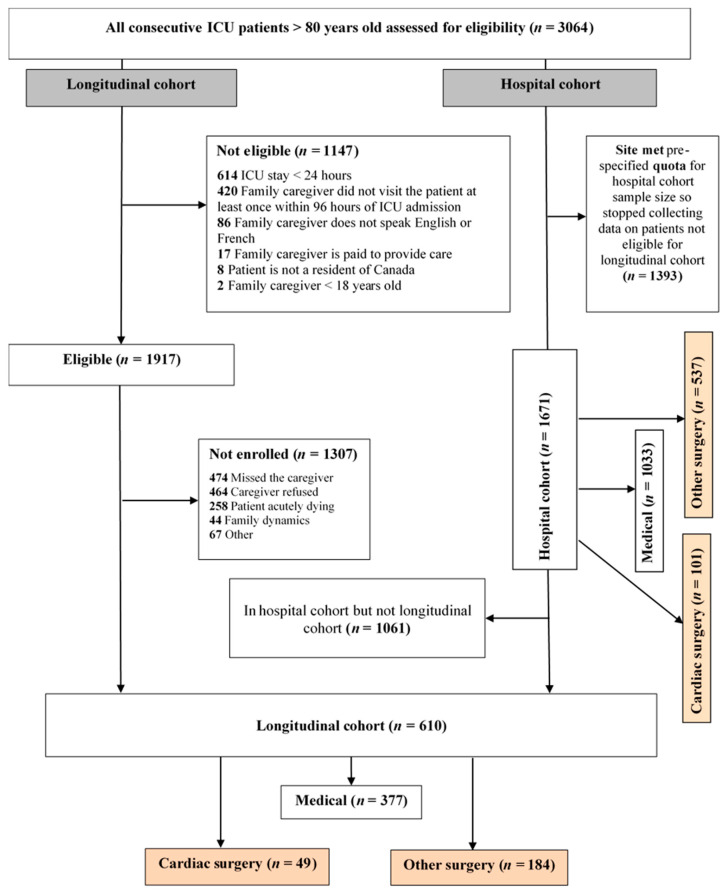
CONSORT Flow diagram, ICU = intensive care unit, CABG = coronary artery bypass graft.

A total of 3064 patients were screened, 610 (377 medical; 49 CXS and 184 surgical ICU) patients were enrolled in the longitudinal cohort and 1671 (1033 medical; 101 CSX and 537 surgical ICU) patients were included in the hospital cohort, (Figure 1). Patients enrolled into the CSX-group had a primary admission diagnosis of valvular heart surgery, coronary artery bypass graft (CABG), or a combination of both. In the CSX-group, there were 67.3% elective and 32.7% emergency surgeries. 69% received low-risk heart surgery (CABG only or valve-only).

### 3.2. Baseline Characteristics of Patients before ICU Admission

To identify potential differences between CSX and surgical ICU patients, baseline characteristics were compared and illustrated in Table 1 (Table A1 for the hospital cohort). At ICU-admission, the SOFA score was significantly higher in the CSX-patients than in the other surgery patients (*p* < 0.001) and CSX-patients had higher APACHE II scores (*p* = 0.03). CSX patients were significantly more often admitted electively to the hospital and/or ICU.

Of all surgical patients, 102 (43%) were considered vulnerable or frail. The baseline characteristics including age, co-morbidities, frailty, and prior living status were similar between groups.

### 3.3. Process of Care

Elderly patients undergoing CSX had documented their preferred medical care significantly more often than other patients (69.4% in the CSX-group vs. 41.3% in the surgical ICU-group, *p* < 0.001). Both withholding (6.1% in the CSX-group vs. 24.5% in the surgical ICU-Group, *p* = 0.005) and active withdrawal (2.0% in the CSX-group vs. 8.2% in the surgical ICU-Group, *p* = 0.13) from life-support occurred less frequently in elderly CSX-patients compared to surgical ICU patients, regardless if they underwent scheduled or emergency surgery.

Almost all elderly CSX-patients received life-sustaining treatments, such as vasopressors (85.7%) and invasive ventilation (93.9%), which was significantly more often than surgical ICU patients (56.0% vasopressors and 83.2% invasive ventilation, *p* < 0.001 and *p* = 0.06). However, elderly CSX-patients who required vasopressors or invasive ventilation remained on them for less time than surgical ICU patients (*p* = 0.03 and *p* < 0.001 respectively).

### 3.4. Clinical Outcomes

As shown in Table 2, elderly CSX-patients had significantly shorter index and total ICU-LOS, as well as shorter hospital-LOS (*p* < 0.001) and were less frequently readmitted to the ICU (*p* = 0.24). Table 2 also compares the hospital discharge disposition between the CSX and surgical ICU patients (*p* = 0.21). Elderly CSX-patients had a significantly lower hospital and 12 month-mortality (*p* < 0.001). Results were similar in the larger hospital cohort (Table A2).

**Figure 2:**
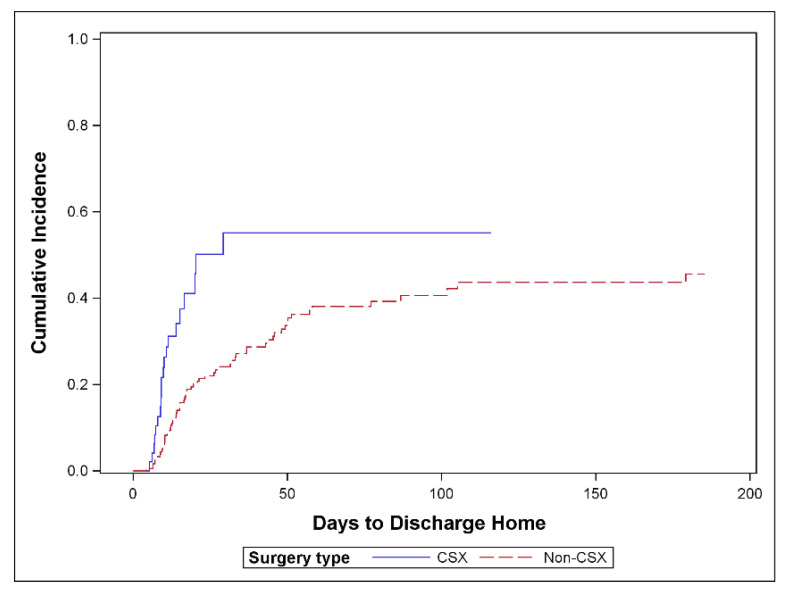
Cumulative incidence of discharge home comparing cardiac surgery versus surgical ICU patients; *n* = 233 (*p* = 0.001); 83 patients discharged home, 74 competing events and 76 censored, CSX = cardiac surgery patients, non-CSX: surgical ICU patients.

Figure 2 shows the cumulative incidence of TTDH by CSX admission, and demonstrates that CSX patients were discharged home at a higher rate than surgical ICU patients (*p* = 0.001).

### 3.5. Influence of Frailty on Patient Outcome

**Figure 3:**
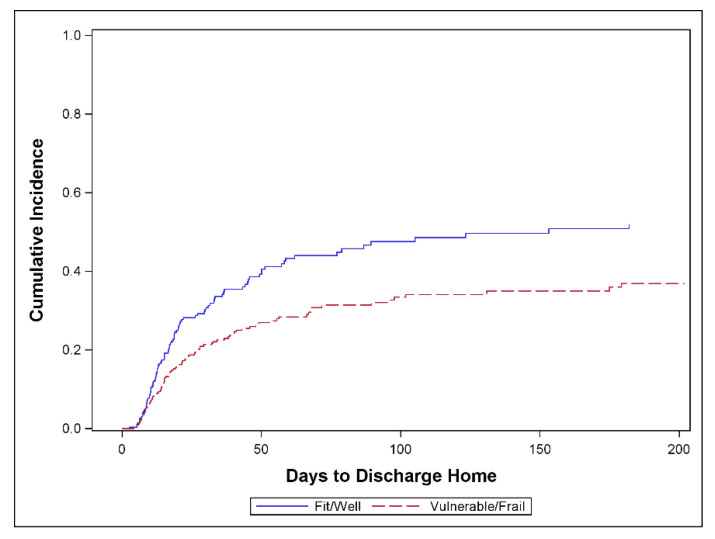
Cumulative incidence of discharge home comparing frail versus non-frail patients when Clinical Frailty Scale (CFS) is dichotomized as vulnerable/frail (CFS ≥ 4) vs. fit/well (CFS ≤ 3). All surgical ICU patients, *n* = 232 (*p* = 0.007); 82 patients discharged home, 74 competing events, and 76 censored. One additional patient was excluded due to unknown CFS data.

**Figure A1:**
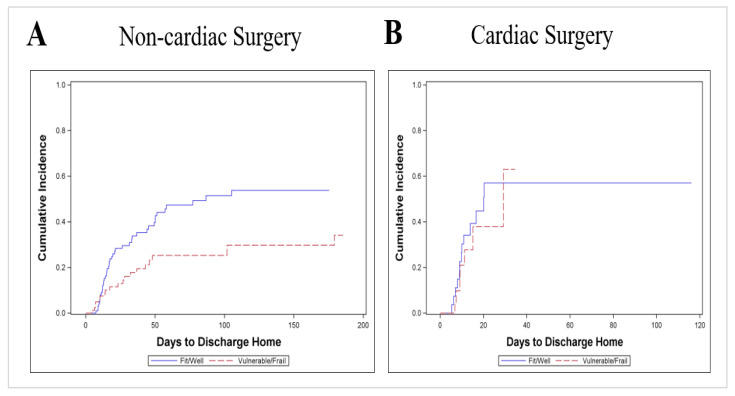
Cumulative incidence of discharge home comparing frail versus non-frail patients when CFS is dichotomized as vulnerable/frail (CFS ≥ 4) vs. fit/well (CFS ≤ 3); CSX = cardiac surgery. (**A**): Surgical ICU patients, *n* = 183 (*p* = 0.006); 62 patients discharged home, 69 competing events and 52 censored; (**B**): CSX-patients *n* = 49 (*p* = 0.61); 20 patients discharged home, 5 competing events, 24 censored.

Figure 3 shows that among all surgical ICU patients, the vulnerable/frail returned home at a lower rate than fit/healthy (*p* = 0.007). The SHR of TTDH for vulnerable/frail vs. fit/well patients was 0.54 (95% CI, 0.34, 0.86). The *p*-value for effect modification between surgery group (CSX vs. surgical ICU patients) and CFS group was not significant (*p* = 0.37) suggesting that the observed difference in the CFS effect between the CSX and surgical ICU patients is consistent with random error. A further subgroup analysis shows that among surgical ICU patients, the SHR of TTDH for vulnerable/frail vs. fit/well patients was 0.49 (95% CI, 0.29, 0.83) while the corresponding SHR for CSX patients was 0.77 (0.32–1.88) (see Figure A1 in the Appendix B).

As shown in Table 3, frailty did not impact ICU- and hospital-LOS in either elderly CSX or elderly surgical ICU patients. Among surgical ICU patients, frail patients had increased 12-month mortality (*p* = 0.02) and were significantly more often discharged into dependent locations (*p* = 0.03). Among the elderly CSX patients there was only one hospital death and two deaths after one year, leaving too few to assess the effect of frailty on mortality.

## 4. Discussion

Frailty is a co-factor describing the patient’s vulnerability for stressful events such as surgery and may influence the short- and long-term outcomes of critically ill patients. The impact of frailty on the outcome of ICU patients has been recognized, but less is known about its prevalence and influence in elderly patients (≥80 years) undergoing cardiac surgery. The characteristics of elderly CSX and surgical ICU-patients were evaluated in this prospective cohort study.

Our findings demonstrate that elderly CSX-patients had similar baseline characteristics regarding comorbidities, frailty, and physical function before ICU admission. However, despite these similarities at baseline, elderly CSX had a higher SOFA and APACHE II scores and needed significantly more supportive treatments, such as vasopressors and invasive ventilation at ICU-admission. This predictable need for organ supporting therapies occurs in the majority of CSX patients during the early recovery phase as a result of myocardial ischemia/reperfusion injury and major surgical trauma and thus may even be more pronounced when compared to surgical ICU patients, which are not exhibited to the consequences of myocardial ischemia/reperfusion injury. Regarding the surgical ICU patients, almost half of them were considered vulnerable or frail. Frail surgical patients experienced a significantly increased the time to discharge home when compared to otherwise fit/well patients. These overserved effects of frailty remained comparable to CSX patients, suggesting that the observed difference in the CFS effect between the CSX and surgical ICU patients remains consistent. A further subgroup analysis demonstrated that among surgical ICU patients, the subdistribution hazard ratio of time to discharge home for frail vs. fit/well patients was significantly reduced, whereas no significant influence could be detected for CSX patients.

Cardiac surgery can trigger an acute and severe systemic inflammatory reaction that leads to relevant organ dysfunctions, including myocardial dysfunction with resulting major hemodynamic alterations during and after surgery [6]. This reaction is typically limited to the first few hours after surgery and may be followed by a recovery with stabilization of hemodynamics and organ function. The postoperative decrease of systemic inflammation in parallel to the recovery from myocardial stunning is also reflected by the patients’ overall shorter ICU- and hospital-LOS, as well as lower ICU-readmission-rate, higher hospital-discharge rate, and significantly lower hospital and 12-month mortality. Since life-supporting treatments are standard in the postoperative care of CSX patients, it would be important to preoperatively identify those patients—especially elderly patients—at a high risk for prolonged need of life support. These “high-risk patients” might be a target group for a preoperative optimization and prehabilitation strategies, such as treatment of malnutrition and sarcopenia [17,18]. In the surgical as well as medical ICU patients, prehabilitation might not be possible due to the low percentage of expected ICU admissions (roughly 27% in surgical and 2% in patients admitted for all causes except cardiac surgery). In the group of CSX patients however, this may be an attractive and feasible (roughly two-thirds of patients underwent elective cardiac surgery) opportunity to improve patients’ long-term outcomes.

Frail patients with their limited reserves are expected to be more vulnerable to disturbances of homeostasis [14,15], and thus it is increasingly felt that frailty might lead to worse outcomes in patients undergoing cardiac surgery. However, in this analysis from a multicenter observational study, frailty did not significantly impact ICU- and hospital-length of stays, or the discharge location in either of the patient groups. Among surgical ICU patients, frailty was associated with significantly increased 12-month mortality and increased rate of discharge to a dependent location, but the CSX sample size was too small to reliably assess these associations. In this context, our study results highlight that mortality alone may not be a sensible outcome parameter to detect the influence of frailty in CSX patients. Patient-centered outcomes, such as muscle strength, physical function, and quality of life might be more sensitive and useful in this case and increasingly used in current studies [4,12,17]. In the same vein, the use of CFS may not be a sufficiently granular frailty assessment tool to predict outcome in this patient cohort, especially as most patients were classified as CFS 3 (managing well) or CFS 4 (vulnerable). Perhaps the use of a different tool for the assessment of frailty would allow a more detailed judgement. To determine preoperative risk factors, more comprehensive research with larger groups of patients, especially higher-risk cardiac surgery patients is required. These patients could represent targets for specific prehabilitation strategies [17,18].

The results of this trial are not confirmed in two meta-analyses, which both showed a strong relationship between frailty and patient outcome in cardiac surgery patients [30,31]. In addition, other prospective cohort studies in elderly patients undergoing cardiac surgery have shown physical function to be an independent predictor of mortality, which might be due to selection of different patients or larger sample sizes [32,33].

### Limitations

Our study has several limitations. First, this is an explorative study, which should be interpreted carefully. The small number of CSX patients in the study and respectively in the frail vs. non-frail categories might have limited our findings. Furthermore, the discrepancy in the group sizes may restrict conclusions. In the same vein, the inclusion criteria of our study (aged over 80 and ICU stay) led to a heterogeneous patient cohort, limiting conclusions drawn from our data.

Second, in the CSX group, the present data is most likely influenced by rather low-risk patients receiving mostly elective CABG or valve surgery. This is reflected in the rather short ICU-stay and low overall mortality. Therefore, our findings may not be widely applied to all patients undergoing cardiac surgery, for example patients undergoing emergency or complex heart surgeries. Patients too sick to receive open heart surgery were not included in the CSX-group, leading to selection bias of the patients. In the same vein, this study compares octogenarians admitted to the ICU therefore diverse surgical patients, adding considerable heterogeneity to the study population, and complicating the interpretation of the scores used (e.g., APACHE II and SOFA Score). The comparison between these patients could nevertheless be important for clinicians to I) better select patients whom an open cardiac surgery is offered, II) allow for preoperative optimization of comorbidities in elective cardiac surgeries and III) better understand the dynamics of critical illness in cardiac surgery patients in comparison to other elderly ICU patients. Taken together, knowing the characteristics of these patients in comparison to other ICU patients may help the clinician to focus on important aspects of patient care for each individual patient.

Another important component not captured in this study is the length of hospital stay before ICU admission. A long preoperative hospital stay negatively impacts muscle strength and physical and cardiocirculatory function in a process called “hospitalization associated disability” [34], as well as nutritional status and mortality [35].

## 5. Conclusions

In conclusion, frailty may be an important factor to consider for assessing risk and predicting outcomes for cardiac surgery patients. Our results may recommend the use of a different, more granular frailty assessment tool to be able to predict outcomes. While the clinical importance of frailty for low risk procedures remains questionable, it needs to be investigated in a group of high-risk cardiac surgery patients. A validated cut-off for frailty has not yet been established. More comprehensive research with larger groups of patients—especially focusing on higher risk cardiac surgery patients—is warranted to determine preoperative risk factors, which could represent targets for specific prehabilitation strategies and to determine if the observed differences can be generalized to all elderly patients undergoing cardiac surgery.

## Figures and Tables

**Table 1:** Baseline characteristics, continuous variables are reported as mean ± standard deviation (minimum–maximum) or median (Q1 to Q3), categorical variables are reported as count (%); Abbreviations: CSX: Cardiac Surgery, SOFA = Sequential Organ Failure Assessment, APACHE = Acute Physiology and Chronic Health Evaluation II, ICU = Intensive Care Unit, * Residential living status prior to hospital admission.

	Longitudinal Cohort		
	CSX (*n* = 49)	Surgical ICU (*n* = 184)	*p*-Values
**Age in years**	83.8 ± 3.1 (80–93)	84.4 ± 3.4 (80–96)	0.33
**Sex**			0.68
**Male**	28 (57.1%)	99 (53.8%)	
**Female**	21 (42.9%)	85 (46.2%)	
**Admission APACHE II Score**	22.6 ± 6.9 (12–34)	20.2 ± 6.5 (7–49)	0.03
**Baseline SOFA**	6.6 ± 2.2 (1–13)	5.0 ± 3.2 (0–14)	<0.001
**Charlson Comorbidity Index**	1 [0 to 3]	2 [0 to 3]	0.62
**Clinical Frailty Scale**	3 [3 to 4]	3.0 [2 to 4]	0.56
Fit/Well (1–3)	28 (57.1%)	102 (55.4%)	
Vulnerable/Frail (4–7)	21 (42.9%)	81 (44.0%)	
Missing	0 (0.0%)	1 (0.5%)	
**Admission type**			<0.001
Surgical elective	33 (67.3%)	50 (27.2%)	
Surgical emergency	16 (32.7%)	134 (72.8%)	
**Primary ICU diagnosis**			<0.001
Cardiovascular/vascular	49 (100.0%)	35 (19.0%)	
Respiratory	0 (0.0%)	10 (5.4%)	
Gastrointestinal	0 (0.0%)	84 (45.7%)	
Neurologic	0 (0.0%)	24 (13.0%)	
Sepsis	0 (0.0%)	2 (1.1%)	
Trauma	0 (0.0%)	10 (5.4%)	
Renal	0 (0.0%)	2 (1.1%)	
Gynecologic	0 (0.0%)	1 (0.5%)	
Orthopedic	0 (0.0%)	16 (8.7%)	
**Ethnicity**			0.54
Asian/Pacific	0 (0.0%)	6 (3.3%)	
African/Black North American	0 (0.0%)	3 (1.6%)	
Caucasian	48 (98.0%)	170 (92.4%)	
East Indian	0 (0.0%)	1 (0.5%)	
Native Canadian	0 (0.0%)	1 (0.5%)	
Other	1 (2.0%)	1 (0.5%)	
Missing	0 (0.0%)	2 (1.1%)	
Living status *			0.63
Alone at home	18 (36.7%)	52 (28.3%)	
With family member at home	18 (36.7%)	71 (38.6%)	
With someone else	10 (20.4%)	36 (19.6%)	
Supervised setting	2 (4.1%)	17 (9.2%)	
Nursing home	1 (2.0%)	7 (3.8%)	
Missing	0 (0.0%)	1 (0.5%)	

**Table 2:** Clinical outcomes in longitudinal and hospital groups, presented as median (Q1–Q3) or number and count (%). Abbreviations: LOS = Length of stay, ICU = Intensive Care Unit.

	Longitudinal Cohort		
	CSX (*n* = 49)	Surgical ICU (*n* = 184)	*p*-Value
**Index ICU LOS (days)**	2.9 (2.0, 6.1)	5.1 (3.0, 9.9)	<0.001
**Total ICU LOS (days)**	2.9 (2.0, 6.1)	5.5 (3.1, 11.1)	<0.001
**Patients with at least one ICU readmission**	2 (4.1%)	17 (9.2%)	0.24
**Total hospital LOS (days)**	12.5 (9.1, 19.7)	24.0 (13.1, 43.8)	<0.001
**Hospital mortality**	1 (2.0%)	41 (22.3%)	0.001
**Discharged from hospital**			0.21
Ward in another hospital	17 (35.4%)	44 (30.8%)	
ICU in another hospital	0 (0.0%)	1 (0.7%)	
Long term care facility	4 (8.3%)	27 (18.9%)	
Home	20 (41.7%)	63 (44.1%)	
Rehab	4 (8.3%)	5 (3.5%)	
Palliative Care	0 (0.0%)	1 (0.7%)	
Other	3 (6.3%)	2 (1.4%)	
**12 months mortality**	4 (8.2%)	66 (35.9%)	<0.001

**Table 3:** Influence of frailty on patient outcome—comparison cardiac surgery versus non-cardiac surgery/surgical ICU patients, Abbreviations: LOS-Length of stay; ICU-Intensive Care Unit, * Independent = home + rehabilitation, # Dependent = hospital, long-term- and palliative care).

	Cardiac Surgery (*n* = 49)			Surgical ICU Patients (*n* = 183)		
	Fit/Well (*n* = 28)	Vulnerable/Frail (*n* = 21)	*p*-Value	Fit/Well (*n* = 102)	Vulnerable/Frail (*n* = 81)	*p*-Value
**ICU-LOS**						
**Index ICU-LOS**	3.5 (2.0, 7.0)	2.8 (2.0, 3.2)	0.28	5.1 (3.0, 10.8)	5.2 (3.0, 9.8)	1.00
**Total ICU-LOS**	3.5 (2.0, 7.5]	2.8 (2.0, 3.2)	0.27	5.6 (3.1, 12.4)	5.5 (3.1, 10.0)	0.81
**Hospital-LOS**	13.2 (9.0, 21.7)	11.0 (9.1, 15.1)	0.48	23.2 (12.9, 44.1)	26.3 (13.6, 43.4)	0.77
**Mortality**						
Hospital	0 (0%)	1 (4.8%)	0.24	20 (19.6%)	21 (25.9%)	0.31
12 months	2 (7.1%)	2 (9.5%)	0.76	29 (28.4%)	37 (45.7%)	0.02
**Discharge location**						
Independent *	14 (50.0%)	10 (47.6%)	1.00	45 (44.1%)	22 (27.2%)	0.03
Dependent #	14 (50.0%)	10 (47.6%)		37 (36.3%)	38 (46.9%)	

## Appendix A. Description of the Hospital Cohort

**Table A1:** Baseline characteristics of the hospital cohort, continuous variables are reported as mean ± standard deviation (minimum–maximum) or median (Q1 to Q3), categorical variables are reported as count (%); Abbreviations: SOFA = Sequential Organ Failure Assessment, APACHE II = Acute Physiology and Chronic Health Evaluation II. * Residential living status prior to hospital admission.

	Hospital Cohort		
	CSX (*n* = 101)	Surgical ICU Patients (*n* = 537)	*p*-Value
**Age in years**	83.4 ± 2.7 (80–93)	84.5 ± 3.4 (80–97)	0.005
**Sex**			0.08
**Male**	63 (62.4%)	284 (52.9%)	
**Female**	38 (37.6%)	253 (47.1%)	
**Admission APACHE II Score**	23.6 ± 7.2 (10–40)	20.8 ± 7.3 (7–49)	<0.001
**Baseline SOFA**	6.7 ± 2.1 (1–13)	4.9 ± 3.2 (0–17)	<0.001
**Charlson Comorbidity Index**	1 (0 to 2)	1 (0 to 3)	0.08
**Clinical Frailty Scale**	Not Collected		
Fit/Well (1–3)			
Vulnerable/Frail (4–7)			
Missing			
**Admission type**			<0.001
Surgical elective	71 (70.3%)	149 (27.7%)	
Surgical emergency	30 (29.7%)	388 (72.3%)	
**Primary ICU diagnosis**			<0.001
Cardiovascular/vascular	101 (100.0%)	95 (17.7%)	
Respiratory	0 (0.0%)	28 (5.2%)	
Gastrointestinal	0 (0.0%)	224 (41.7%)	
Neurologic	0 (0.0%)	72 (13.4%)	
Sepsis	0 (0.0%)	7 (1.3%)	
Trauma	0 (0.0%)	37 (6.9%)	
Renal	0 (0.0%)	8 (1.5%)	
Gynecologic	0 (0.0%)	1 (0.2%)	
Orthopedic	0 (0.0%)	65 (12.1%)	
**Ethnicity**			0.54
Asian/Pacific	0 (0.0%)	6 (1.1%)	
African/Black North American	0 (0.0%)	3 (0.6%)	
Caucasian	48 (47.5%)	170 (31.7%)	
East Indian	0 (0.0%)	1 (0.2%)	
Native Canadian	0 (0.0%)	1 (0.2%)	
Other	1 (1.0%)	1 (0.2%)	
Missing	52 (51.5%)	355 (66.1%)	
**Living status ***			0.63
Alone at home	18 (17.8%)	52 (9.7%)	
With family member at home	18 (17.8%)	71 (13.2%)	
With someone else	10 (9.9%)	36 (6.7%)	
Supervised setting	2 (2.0%)	17 (3.2%)	
Nursing home	1 (1.0%)	7 (1.3%)	
Missing	52 (51.5%)	354 (65.9%)	

**Table A2:** Clinical outcomes in longitudinal and hospital groups, presented as median (Q1–Q3) or number and count (%). Abbreviations: LOS= Length of stay, ICU= Intensive Care Unit.

	Hospital Cohort		
	CSX (*n* = 101)	Surgical ICU Patients (*n* = 537)	*p*-Value
**Index ICU LOS (days)**	2.0 (1.1, 3.7)	3.6 (1.8, 7.6)	<0.001
**Total ICU LOS (days)**	2.0 (1.1, 3.9)	3.8 (1.8, 8.1)	<0.001
**Patients with at least one ICU readmission**	3 (3.0%)	40 (7.4%)	0.10
**Total hospital LOS (days)**	10.1 (7.0, 14.8)	19.5 (9.7, 36.3)	<0.001
**Hospital mortality**	4 (4.0%)	152 (28.3%)	<0.001
**Discharged from hospital**			0.004
Ward in another hospital	33 (34.0%)	106 (27.5%)	
ICU in another hospital	0 (0.0%)	10 (2.6%)	
Long term care facility	7 (7.2%)	73 (19.0%)	
Home	48 (49.5%)	184 (47.8%)	
Rehab	4 (4.1%)	8 (2.1%)	
Palliative Care	2 (2.1%)	1 (0.3%)	
Other	3 (3.1%)	3 (0.8%)	
**12 months mortality**	7 (6.9%)	177 (33.0%)	<0.001
